# Supplementary material for: Adiponectin preserves metabolic fitness during aging
Source: eLife. 2021 Apr 27;10:e65108. doi: 10.7554/eLife.65108 (PMC8099426; doi:10.7554/eLife.65108)
Supplement: Supplementary file 1. — Table displaying the sample size, statistical test method, and p-value for the list figures. [file elife-65108-supp1.docx]

**Supplementary Files**

**Supplementary File 1. Statistical Information**

| **Figure** | **N(sample size)** | **Statistical test method** | ***p*-value** |
| --- | --- | --- | --- |
| Figure 1A | WT: 81  APN-KO: 72 | Log-rank (Mantel-Cox) test | *p*=0.0435 |
| Figure 1B | WT: 63  APN-KO: 60 | Log-rank (Mantel-Cox) test | *p*=0.0002 |
| Figure 2A | WT: 32  APN-KO: 43 | Unpaired *Student’s t* test | Week110: *p=*0.0039 |
| Figure 2D | WT: 8  APN-KO: 7 | Unpaired *Student’s t* test | Time 15’: *p=*0.0084  Time 30’: *p=*0.0077 |
| Figure 2E | WT: 9  APN-KO: 10 | Unpaired *Student’s t* test | 1h: *p=*0.0498  2h: *p=*0.0027 |
| Figure 2F | WT: 8  APN-KO: 8 | Unpaired *Student’s t* test | 2h: *p=*0.0064 |
| Figure 2H | WT: 8  APN-KO: 7 | Unpaired *Student’s t* test in each time point | *Day1*: *Minimum p=*0.0010  *Day2*: *Minimum p=*0.0060  *Day3*: *Minimum p=*0.0244  *Day4*: *Minimum p=*0.0089 |
| Figure 4A | NCD  WT:10  APN-KO:10  HFD:  WT:10  APN-KO:10 | Unpaired *Student’s t* test | *Tnf-* α NCD: *p=0.0377*  *Il1b* NCD: *p=* 0.0024  *F4/80* NCD*: p=0.0015*  *Mcp-1* NCD*: p=0.0085*  *Crp* NCD*:p=0.0019*  *Tnf-* α HFD: *p=0.0201*  *Il1b* HFD: *p=* 0.0502  *F4/80* HFD*: p=0.2100*  *Mcp-1* HFD*: p=0.0466*  *Crp* HFD*:p=0.0176* |
| Figure 4B | NCD  WT:8-10  APN-KO:8-10  HFD:  WT:8-10  APN-KO:8-10 | Unpaired *Student’s t* test | *Tnf-* α NCD: *p=0.0486*  *Il1b* NCD: *p=0.0215*  *F4/80* NCD*: p=0.0136*  *Mcp-1* NCD*: p=0.0027*  *Crp* NCD*:p=0.0336*  *Tnf-* α HFD: *p=0.0055*  *Il1b* HFD: *p=* 0.0119  *F4/80* HFD*: p=0.0246*  *Mcp-1* HFD*: p=0.0495*  *Crp* HFD*:p=0.0767* |
| Figure 4C | HFD:  WT:3  APN-KO:3 | Unpaired *Student’s t* test | Total macrophage:p=0.0209  Kuffer cell:p=0.0076 0.1348  Monoctes-derived macrophage:  p= 0.1348 |
| Figure 4D | Old  WT: 6 APN-KO:6  Young:  WT:5 APN-KO:5 | Unpaired *Student’s t* test | *Il6 Old*: *p=*0.0200  *Il6 Young:*  *p=*0.5250  *Tnf-* α *Old*:  *p=*0.0225  *Tnf-*α *Young:*  *p=*0.8918  *Tnf-*α *WT：*  *p=*0.0154  *Col1a1 Old:*  *p=*0.0382  *Col1a1 Young:*  *p=*0.0381  *Col1a1 WT:*  *p=*0.0007  α*Sam Old:*  *p=*0.0271  α*Sam Young:*  *p=*0.7808  α*Sam WT:*  *p=*0.0012 |
| Figure 4E | WT: 6  APN-KO: 6 | Unpaired *Student’s t* test | AST: *p=*0.0134  ALT: *p=*0.0073 |
| Figure 4G | WT: 6 or 10  APN-KO:10 | Unpaired *Student’s t* test | Kidney Cdkn2a:p=0.0013  Kidney Glb-1:p=0.0083  Liver Cdkn2a:p=0.046  Liver Glb-1:p=0.1228 |
| Figure 4H | WT: 7-10  APN-KO:8-10 | Unpaired *Student’s t* test | Kidney Cdkn2a:p=0.3338  Kidney Glb-1:p=0.0112  Liver Cdkn2a:p=0.4892  Liver Glb-1:p=0.0245 |
| Figure 5A | Control: 45  ΔGly: 37 | Log-rank (Mantel-Cox) test | *p=*0.7048 |
| Figure 5C | Glucose and Insulin  Control: 7 ΔGly: 8  IGF-1:  Control: 10ΔGly: 9 | Unpaired *Student’s t* test | Fasting Glucose: *p<*0.0001  Fasting Insulin: *p=*0.0017  *IGF-1: p=0.0015* |
| Figure 5D | Control Islets: 54  ΔGly Islets: 58 | Unpaired *Student’s t* test | *p*=0.0012 |
| Figure 5E | Control: 8  ΔGly: 8 | Unpaired *Student’s t* test | Time0’: *p<*0.0001  Time120’:  *p*=0.0132 |
| Figure 5F | Control: 7  ΔGly: 8 | Unpaired *Student’s t* test | Time0’: *p=* *0.006*  Time15’:  *p*=0.05 |
| Figure 5G | Control: 8  ΔGly: 8 | Unpaired *Student’s t* test | Time15’: *p=0.0014*  Time30’: *p=*0.0012  Time60’: *p=0.0566*  Time90’: *p=0.0137*  Time120’: *p=*0.5305 |
| Figure 5H | Control: 8  ΔGly: 9 | Unpaired *Student’s t* test | 2h*: p=*0.0127  6h*: p=*0.0247 |
| Figure 5I | Control: 8  ΔGly: 9 | Unpaired *Student’s t* test | *p=*0.0138 |
| Figure 5J | Control: 8  ΔGly: 9 | Unpaired *Student’s t* test | Time 0’: *p=*0.0476 |
| Figure 6C | Control: 8  ΔGly: 6 | Unpaired *Student’s t* test | *SAT: p=*0.2244  *WAT: p=*0.0042 |
| Figure 6F | Control: 8  ΔGly: 6 | Unpaired *Student’s t* test | *F4/80: p=*0.0216  *Il1b: p=*0.0618  *Mcp: p=*0.0318  α*Sam: p=*0.0714  *Col1a1: p=*0.0383  *Col3a1: p=*0.0198 |
| Figure 6G | Control: 5  ΔGly: 5 | Unpaired *Student’s t* test | Corticosterone*: p=0.0003* |
| Figure 1-figure supplement 1C | WT: 4 APN-KO: 4  Control: 4 ΔGly: 4 | Unpaired *Student’s t* test | *WT&APN-KO:*  *p<*0.0001  *WT&* ΔGly*: p=*0.0052 |
| Figure 2-figure supplement 1A | NCD  WT:5  APN-KO:5  HFD:  WT:5 or 7  APN-KO:5 | Unpaired *Student’s t* test | *Fat mass NCD:p=0.2347*  *Lean mass NCD:p=0.3571*  *SAT NCD:p=0.0048*  *WAT NCD:p=0.0804*  *BAT NCD:p=0.9056*  *Fat mass HFD:p=0.0509*  *Lean mass HFD:p=0.1000*  *SAT HFD:p=0.3646*  *WAT HFD:p=0.4305*  *BAT HFD:p=0.8378* |
| Figure 2-figure supplement 1B | NCD  WT:5  APN-KO:5 | Unpaired *Student’s t* test | *TMC NCD:p=0.1095*  *BMD NCD:p=0.8645*  *Spinal BMD:p=0.1151*  *Femoral BMD:p=0.2356* |
| Figure 2-figure supplement 1C | HFD:  WT: 7  APN-KO:4 | Unpaired *Student’s t* test | *TMC HFD:p=0.0005*  *BMD HFD:p=0.0099*  *Spinal HFD:p=0.0038*  *Femoral HFD:p=0.0001* |
| Figure 2-figure supplement 1E | WT: 5  APN-KO: 6 | Unpaired *Student’s t* test | Kidney weight*:p=*0.0416 |
| Figure 4-figure supplement 1A | WT:7  APN-KO:7 | Unpaired *Student’s t* test | *Il6: p=*0.0245  *Tnf-* α*: p=*0.0106  *F4/80: p=*0.0324  *Il1b: p=*0.0006  *Mcp: p=*0.0027  α*Sam: p=*0.2525  *Col1a1: p=*0.1531  *Col3a1: p=*0.0132 |
| Figure 4-figure supplement 1B | WT: 5  APN-KO: 5 | Unpaired *Student’s t* test | NCD*: p=0.0539*  HFD*:p=0.0741* |
| Figure 4-figure supplement 1C | WT: 6  APN-KO: 6 | Unpaired *Student’s t* test | *Il1b Hepatocyte: p=0.4220*  *Il1b Macrophage: p=0.0366*  *Tnf-* α *Hepatocyte: p=0.9936*  *Tnf-* α *Macrophage: p=0.0015*  *CD11b Hepatocyte: p=0.8656*  *CD11b Macrophage: p=0.7710*  *F4/80 Hepatocyte: p=0.6995*  *F4/80 Macrophage: p=0.0545*  *CD206 Hepatocyte: p=0.1125*  *CD206 Macrophage: p=0.0128* |
